# New approaches in pear preservation: Putrescine and modified atmosphere packaging applications to maintain fruit quality during cold storage

**DOI:** 10.1002/fsn3.4290

**Published:** 2024-06-25

**Authors:** Ferhat Ogurlu, Emine Kucuker, Erdal Aglar, Ceyda Kizgin Ozcengiz, Cuneyt Uyak

**Affiliations:** ^1^ GAP International Agricultural Research and Training Center Diyarbakir Turkey; ^2^ Faculty of Agriculture, Department of Horticulture Siirt University Siirt Turkey; ^3^ Faculty of Agriculture, Department of Horticulture Van Yüzüncü Yıl University Van Turkey

**Keywords:** antioxidant capacity, fruit firmness, titratable acidity, total phenolics, weight loss

## Abstract

Pear (*P. communis* L.), which is a climacteric fruit species, has a very short storage and shelf life, and significant losses occur due to high metabolic activity and the fruit's respiration rate after harvest. Therefore, preventing or reducing post‐harvest quality losses in pear is one of the most basic problems awaiting solution. In this study, we planned for this purpose; the fruits of the Ankara pear cultivar treated with modified atmosphere packaging **(**MAP), putrescine (1 mM), and MAP + putrescine were stored for 120 days at 1°C and 90 ± 5% relative humidity. The quality analyses and measurements, such as weight loss, decay rate, fruit firmness, soluble solids content (SSC), titratable acidity (TA), total phenolic compounds, antioxidant capacity, organic acids, and specific phenolic compounds, were performed on the 30th, 60th, 90th, and 120th days. Weight loss and decay ratios were lower for putrescine and putrescine + MAP‐applied fruit. With these applications, the softening of the fruit was slowed down, and the increasing SSC in the fruit and the decreasing TA rates were lower, and thus the ripening of the fruit was delayed. Changes in individual phenolic content and organic acids were lower in MAP and putrescine‐applied fruit. The study revealed that MAP and putrescine applications in pear can be used effectively to maintain fruit quality after harvest.

## Graphical abstract

Putrescine and MAP treatments reduced the storage losses and decay ratio during cold storage in pear. Putrescine and MAP treatments maintained the fruit quality and bioactive compounds of pear during cold storage. Putrescine and MAP treatments have a significant effect on extending the post‐harvest life of pear.

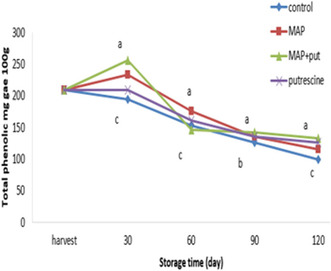

## 1 INTRODUCTION

In pear, which is a climacteric fruit species, the storage and shelf life are quite short, and significant losses occur due to the high metabolic activity and fruit respiration rate after harvest (Huang et al., 2022). Therefore, preventing or reducing post‐harvest quality losses in pear is one of the most fundamental problems awaiting solution. The quality deterioration caused by high respiration, evaporation, and pigment degradation is one of the critical problems affecting the storage ability and shelf life of pear (Li et al., 2021). Many studies have been carried out with different applications to delay ripening, maintain storage quality, and control post‐harvest decay by slowing down respiration and pigment degradation in pear (Huang et al., 2022; Latt et al., 2023). MAP application, which can change the gas concentration around the fruit and create a suitable atmosphere to limit respiration and metabolic activity by affecting humidity rates, can provide significant advantages in fruit storage (Petracek et al., 2002) by reducing water loss, respiration activity, ethylene production, enzymatic reactions, and physiological changes and by maintaining the fruit flesh firmness (Moradinezhad & Dorostkar, 2021). In previous studies, it was reported that MAP application affected fruit quality after harvest in fruit species such as kiwifruit (Hertog et al., 2004), sweet cherry (Aglar et al., 2017), pomegranate (Candir et al., 2018), jujube (Islam et al., 2022), cornelian cherry (Ozturk & Aglar, 2019), and plum (Avci et al., 2023).

Polyamines have a significant role in many biological processes such as cell division, cell elongation, fruit development and maturation, and the response to biotic and abiotic stress in plants (Janne et al., 2004). Putrescine, which delays the removal of the epicuticular waxes that protect the membrane integrity of the fruit and play an important role in water exchange from the peel, can be used to extend the storage period and shelf life. In order to preserve post‐harvest quality in pear, the MAP (Wang et al., 2020) application has been carried out. However, there is no study on the combination of putrescine and MAP. The study is aimed at determining the effect of putrescine and MAP applications on post‐harvest fruit quality in the “Ankara” pear cultivar.

## 2 MATERIALS AND METHODS

The plant material (fruit) for this research was harvested from trees of the “Ankara” pear cultivar grafted onto quince A rootstock. The fruit harvested at commercial maturity was placed in 10 kg capacity plastic boxes (Plastas, Türkiye) and quickly transferred to the GAPUTAEM Quality and Technology Laboratory with a refrigerated vehicle (12 ± 1.0°C and 75 ± 5.0% relative humidity). The bruised or damaged fruit were eliminated and excluded from evaluation. The fruits selected as examples in terms of color and size were divided into 4 groups. 1: The fruit stored without any application (control), 2: The fruit stored by placing them in Xtend® modified atmosphere bags (MAP application), 3: The fruit dipped in 1 mM putrescine solution for 10 min and stored after drying at room conditions for 20 min (Putrescine application), and 4: The fruit dipped in 1 mM putrescine solution for 10 min and dried under room conditions for 20 min and then placed in Xtend® modified atmosphere bags (Putrescine + MAP application). In fruit, which were stored at 0 ± 0.5°C and 90 ± 5% RH for 120 days, the measurements and analyses were made in three replicates on the 30th, 60th, 90th, and 120th days of storage, and 5 fruits were used for each repetition.

### 2.1 Weight loss

After the fruits were applied with putrescine, the total weight (Wi) of the fruits for each application was measured with a digital scale (Radwag, Poland) with an accuracy of 0.01 g. In fruits stored for 120 days, the fruit weight (Wf) measurements were performed again on the 30th, 60th, 90th, and 120th days of the cold storage. The weight loss occurring at applications and measurement periods was determined using the following Equation (1).

(1) $$\mathrm{WL}=\frac{W_{i}-W_{f}}{W_{i}}\times 100$$

### 2.2 Decay rate

After the putrescine application, the number of fruits in each replicate (5 fruits) and the total number of fruits for each application (15 fruits) were recorded (TF). In fruits stored for 120 days, on the 30th, 60th, 90th, and 120th days of the cold storage, the rotten fruits in each repetition and in each application were detected by including the rotten fruits in those with mycelial development on the peel. The decay rate (DR) in the treatments was recorded as a percentage using the following Equation (2).

(2) $$\mathrm{DR}=\frac{\mathrm{TF}‐\mathrm{DF}}{\mathrm{TF}}\times 100$$

### 2.3 Fruit firmness

In each measurement period, the fruit flesh firmness for each repetition (5 fruits) and each application (15 fruits) was calculated as N by cutting the fruit skin from two opposite points and determining it with an effegi penetrometer (FT‐327; McCormick, WA, ABD) with a 7.9 mm tip.

### 2.4 Soluble solid content and titratable acidity

The fruits, which were washed with distilled water during the measurement periods, were decomposed, made homogeneous, and then passed through a cheesecloth to obtain fruit juice to be used in SSC and TA measurements. The SSC was measured using a digital refractometer (Atago PAL‐1, USA) and recorded as a percentage (%). To determine TA, 10 mL of the obtained fruit juice was taken, and distilled water was added to the amount taken (10 mL). A sufficient amount of 0.1 N sodium hydroxide (NaOH) was added to raise the pH of the solution to 8.2. TA was determined by taking into account the amount of NaOH spent in the titration process and recorded as g malic acid kg^−1^.

### 2.5 Total phenolics and antioxidant capacity

The fruits, which were washed with distilled water during the measurement periods, were decomposed and made homogeneous; 30 mL of the obtained homogenate was taken and placed in 50‐mL falcon tubes and kept at −20°C until the analysis period. Before starting the analyses, the frozen samples were kept at room temperature (21°C) to thaw until they were suitable for analysis. To separate the pulp and juice, the homogenate was centrifuged at 12,000 ***g*** for 35 min at 4°C, and by using the obtained solution, total phenolics and antioxidant capacity were determined by UV–Vis spectrophotometer (Shimadzu, Kyoto, Japan) (Ozturk et al., 2019).

### 2.6 Organic acids

The method reported by Bevilacqua and Califano (1989) was modified, and organic acids were extracted from fruit samples. To homogenize the fruit samples, 10 g of the sample was placed in centrifuge tubes, and 10 mL of 0.009 N H_2_SO_4_ was added, mixed for 1 h, and centrifuged at 630 g for 15 min. The liquid (supernatant) remaining at the top of the centrifuge tube was filtered through filter paper, then passed through a 0.45 μm membrane filter, and finally through the SEP‐PAK C18 cartridge. It was injected into the HPLC (Agilent HPLC 1100 series G 1322 A, Germany) device, and the separations were performed on the appropriate column (Aminex HPX ‐ 87 H, 300 mm × 7.8 mm). Organic acids were determined at wavelengths of 214 and 280 nm. A 0.009 N H_2_SO_4_ solution was used as the mobile phase.

### 2.7 Specific phenolic compounds

To extract the fruit samples, 1 g of homogeneous fruit samples was taken, placed in the test tube, and treated with 5 mL of methyl alcohol. The resulting extract was analyzed by high‐pressure liquid chromatography (HPLC) (Perkin‐Elmer series 200, Norwalk, USA). The HPLC system was equipped with a UV detector (Series 200, UV/Vis detector) and a quaternary solvent distribution system (Series 200, analytical pump) and was used at 280 nm. Analytes were separated by a Phenomenex Kromasil (Phenomenex, Torrance, USA) 100A C18 (250 mm × 4.60 mm, 5 μm) column. The clone temperature was maintained at 26°C using a water bath (Wisebath, WB‐22, Daihan Scientific, Seoul, Korea). The mobile phase was formed from water and acetonitrile (A) containing 2.5% formic acid (B). The mobile phase flow rate was maintained at 1 mL per minute, and 20 μL of the sample was injected and expressed in g 100 g^−1^ in light of the results of the peak areas obtained (Ozturk et al., 2019).

### 2.8 Statistical analysis

The data, whose normal distribution suitability was determined by the Kolmogorov‐Simirnov test and whose homogeneity was checked by the Levene test, were evaluated by analysis of variance. The significance level between applications was determined by Tukey's multiple comparison test. The significance level in interpreting the statistical results performed with the SAS package program (SAS 9.1 version, USA) is *α* = 5%.

## 3 RESULTS AND DISCUSSION

### 3.1 Weight loss, decay ratio, and fruit firmness

**FIGURE 1:**
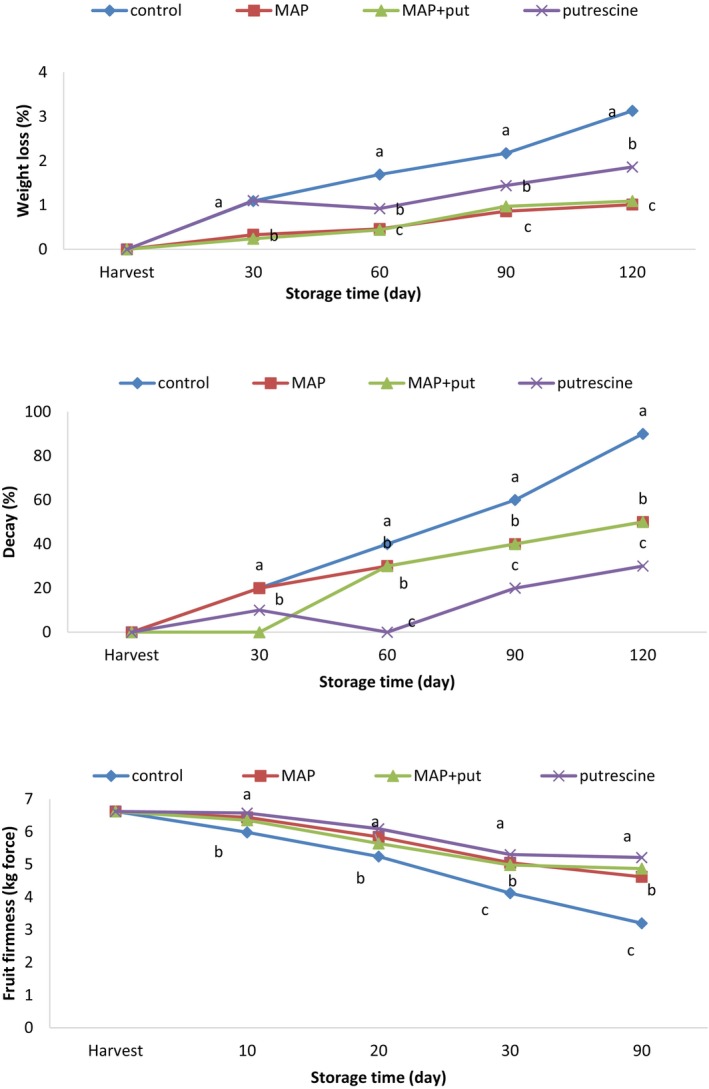
The effect of putrescine and MAP applications on weight loss, decay ratio, and fruit firmness of pear at cold storage. The means in columns with the same letter do not differ according to Tukey's test at *p* < .05.

Weight loss occurs in fruit as a result of water evaporation through transpiration (Kader & Yahia, 2011) and increases in proportion to storage time (Ozturk et al., 2021), causing significant economic damage (Sandhya, 2010). Ben‐Yehoshua and Rodov (2002) reported that a weight loss of 3%–10% may occur in fruit after harvest, and Lownds et al. (1993) suggested that this weight loss is due to water loss from transpiration and respiration, which may vary based on the cuticle and physical properties of the fruit. The weight loss can be reduced by modified atmosphere packaging, which causes reduced respiration in the fruit (Ozturk et al., 2020), and polyamines such as spermidine and putrescine, which delay the degradation of the cell wall in the fruit (Rastegar et al., 2020). The weight loss, which increased with storage time, was lower with MAP application. The lowest weight loss was recorded at the MAP application with 1.08% and the Putrescine + MAP (Put + MAP) application with 1.09%, and the highest weight loss was recorded at the control application with 3.42%. There was no difference between control and putrescine applications on the 30th day; weight loss was lower in putrescine‐applied fruit at other measurement dates. Putrescine applications have an effect on reducing weight loss, and MAP and Put + MAP applications are the most effective in reducing weight loss (Figure 1). Kablan et al. (2008) suggested that carbon atom loss in the respiration cycle can lead to weight loss. Polyamines can reduce respiration by inhibiting ethylene biosynthesis or reducing metabolic rates, thus leading to a significant reduction in weight loss (Champa et al., 2014). Patel et al. (2019) reported that weight loss in pepper was reduced by polyamine application, and the effect varied based on the application dose. It has been reported that the weight loss and decay rate that occur during cold storage in grapes are suppressed by the application of putrescine. The lower weight loss in polyamine‐applied fruit can be attributed to the stabilization and consolidation of both cell integrity and permeability of tissues (Champa et al., 2014). Kibar et al. (2021) reported that putrescine application in peaches reduced the weight loss of peach fruit throughout storage, which may be due to the lower respiration rate in putrescine‐applied fruit.

Fruit decay, which increases in relation to weight loss in post‐harvest storage (Cantín et al., 2011), varies based on fruit species and cultivar. Polyamines, which function as a signaling molecule in plants’ defense mechanisms (Yamakawa et al., 1998) and MAP applications, which regulate respiration rate by changing the gas atmosphere around the fruit (Ozturk et al., 2020), have the potential to control the disease severity in fruit by preventing fungal rot in the fruit and reducing fruit softening during cold storage. During cold storage, the highest rotting rate was detected in the fruit of the control application. The highest decay rate (90%) was recorded for control application, and the lowest (30%) was recorded for putrescine‐applied fruit. There was no difference between control and MAP applications on the 30th day and between Put + MAP and MAP applications on the 60th, 90th, and 120th days (Figure 1). Champa et al. (2014) suggested that polyamines are conjugated to phenolic compounds and hydroxycinnamic acid amides and have a good correlation between the accumulation of hydroxycinnamic acid amides and pathogen resistance. Champa et al. (2014), Khosroshahi and Esna‐Ashari (2007), and Shiri et al. (2013) have reported that the weight loss and decay rate that occurs during storage were suppressed by polyamine applications such as spermidine and putrescine, and the weight loss and decay rate were lower in fruit treated with polyamines. Martinez‐Romero et al. (2006) reported that putrescine application reduced rotting damage compared to control fruit, and Khosroshahi and Esna‐Ashari (2007) reported that putrescine‐applied strawberry fruits were suitable to be offered in the market after 12 and 14 days of storage. Previous studies have shown that polyamine applications reduced the damage of rotting and chilling, and the fruit quality was preserved at the cold storage in fruit species including peach (Kibar et al., 2021), pomegranate (Barman et al., 2011), apricot (Koushesh et al., 2012), papaya (Hanif et al., 2020), mango (Jawandha et al., 2012), and mandarin (Ennab et al., 2020).

Fruit softening, which increases with ripening and occurs as a result of structural changes in the pectin matrix leading to loss of cell wall structure (Posé et al., 2015), is an important problem that affects the post‐harvest life of the fruit and the marketing process. Positively charged polyamines strengthen cell walls by cross‐linking to the carboxyl (COO) group of pectic substances in the cell wall and reduce the softening rate during storage by providing wall rigidity (Valero et al., 2002). However, MAP applications that regulate respiration rate by changing the gas atmosphere around fruit (Ozturk et al., 2020) can reduce fruit softening by reducing the respiration rate and water loss in post‐harvest storage. Fruit flesh firmness values varied between 3.20 and 6.67. Fruit firmness decreased with increasing storage time, but this decrease was lower with MAP and putrescine applications. The lowest values during cold storage were recorded in the fruit of the control application, and the fruit firmness values were higher in putrescine‐applied fruit. There was no difference between MAP and putrescine applications on the 30th, 60th, and 90th days, and the firmness values of MAP‐applied fruit were lower than putrescine‐applied fruit (Figure 1). Fruit flesh firmness was maintained by the putrescine application in fruit species such as plum (Khan et al., 2008), peach (Kibar et al., 2021), papaya (Hanif et al., 2020), plum (Serrano et al., 2003), and apricot (Martinez‐Romero et al., 2006).

### 3.2 Soluble solid content and titratable acidity

**FIGURE 2:**
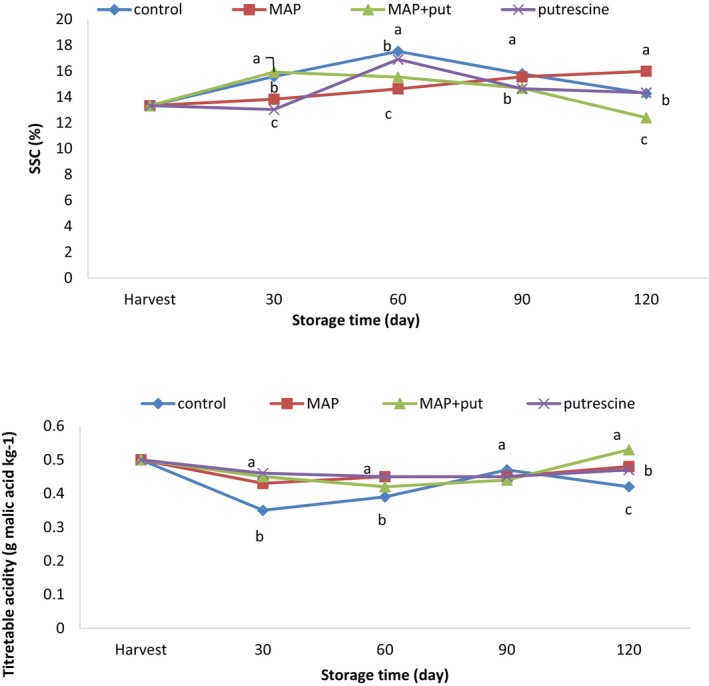
The effect of putrescine and MAP applications on the soluble solid content and titratable acidity of pear at cold storage. The means in columns with the same letter do not differ according to Tukey's test at *p* < .05.

Soluble solid content (SSC) and titratable acidity (TA) are significant quality characteristics that determine the storage period of the fruit (Mahto & Das, 2013). During post‐harvest storage, the acid metabolism causes a decrease in TA and an increase in SSC by converting starch and acid into sugar (Duan et al., 2011). Putrescine and spermidine can prevent spoilage processes in the fruit by polyamine application, which may cause a delay in chemical changes in the fruit during the ripening stage (Jongsri et al., 2017). On the 30th day, the control Put + MAP fruit had higher values than putrescine and MAP application. The highest value was measured in the control fruit on the 60th day. On the 90th day, the higher values were measured in the control and MAP applications, and there was no difference between the two applications. The lowest value was recorded in the Put + MAP application, but there was no difference between the control and putrescine applications. The fluctuations occurred in SSC values during cold storage. TA values decreased during cold storage in all applications. On the 30th day, the lowest TA value was detected in the control application, and there was no difference between putrescine, MAP, and P + MAP applications. Putrescine, MAP, and Put + MAP applications had similar values on the 60th day of cold storage, but there were no differences between all applications in TA values on the 90th day. The highest TA value was measured in the Put + MAP application, and there was no difference between MAP and putrescine applications; the fruit of these applications had a higher TA content than the control group (Figure 2). Putrescine, which delays ripening by reducing ethylene production in fruit, also reduces changes in SSC and TA ratios after harvest (Serrano et al., 2003). Previous studies reported that the change in SSC and TA ratios during cold storage was lower with the putrescine application in fruit species such as plum (Serrano et al., 2003), peach (Kibar et al., 2021), and papaya (Hanif et al., 2020). The increases and decreases in the SSC content during cold storage are based on events such as the transformation of sugar into CO_2_ and H_2_O in parallel with the increase in respiration rate, the transformation of starch into sugar, the increase in dry matter content in parallel with the decrease in the amount of water in the fruit, and the disintegration of polysaccharides in the cell wall (Vieira et al., 2016).

### 3.3 Total phenolics and total antioxidant capacity

**FIGURE 3:**
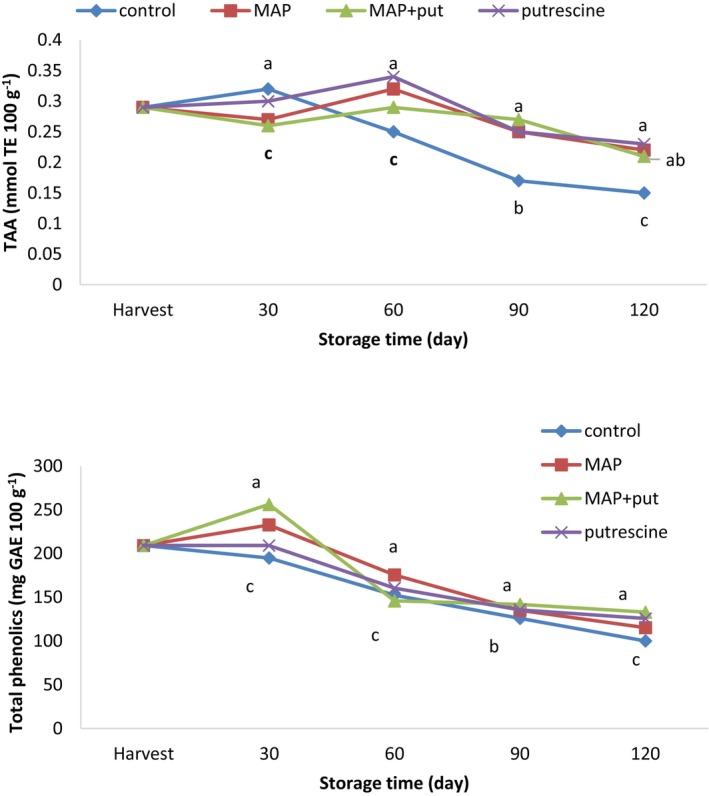
The effect of putrescine and MAP applications on the total phenolics and total antioxidant activity (TAA) of pear at cold storage. The means in columns with the same letter do not differ according to Tukey's test at *p* < .05.

The total phenolic content increased in all applications except the control application on the 30th day, and then it decreased in all applications. At storage, the lowest phenolic substance content was measured in the control application, and the highest phenol content was measured in the Put + MAP application. On the 30th day, the total antioxidant content increased in control and putrescine applications, but decreased in MAP and Put + MAP applications. In the measurements performed on the 60th day, the total antioxidant content decreased in the control group, whereas it increased in the other applications. On the 90th day, the antioxidant content decreased in all applications; at the end of storage, the lowest content was measured in the control and the highest content was measured in the putrescine application (Figure 3). Putrescine application delays the biochemical changes that will occur by preventing ethylene synthesis in the fruit (Abbasi et al., 2019). Jongsri et al. (2017), who suggested phenolic compound content increased during cold storage, reported that the total phenolic content was higher in putrescine‐applied fruit during this process. Similar results were obtained in pomegranate and grape, but it has been reported that the mechanism of action of spermidine is unknown. Serrano et al. (2006) reported that the losses in antioxidant activity and total phenolic content of broccoli decreased with MAP applications. Giacalone and Chiabrando (2013) stated that MAP applications did not have any negative effects on phenolic compounds and antioxidant activity. Khan and Singh (2007) reported that MAP‐applied plums had lower antioxidant activity than control fruit. Similarly, Artés‐Hernández et al. (2006) reported that MAP application delayed the formation of carotenoids and color pigments.

### 3.4 Organic acids and specific phenolic compounds

Polyphenols are secondary metabolites that increase the quality properties, such as firmness, taste, color, and antioxidant properties, of fruit and vegetables and contribute to the plant's defense mechanism (Sreekumar et al., 2014). Polyamines such as melatonin, spemidine, and putrescine, which eliminate reactive oxygen species (ROS) but also induce the gene expression of antioxidant enzymes in plants, increase the level of some beneficial compounds such as natural antioxidants, phenolics, aroma compounds, polyphenols, and soluble solids (Liu et al., 2019). It is used to improve fruit quality (Zhang et al., 2020) and can cause an increase in the content of compounds such as anthocyanins, phenols, and flavonoids (Xia et al., 2020). The decrease in acidity proportional to the ripening period is a natural characteristic (Patel et al., 2019). The amount of organic acids, one of the main substrates required for respiration, decreases as the respiration rate increases. Hormone applications such as polyamine and salicylic acid, which are applied to slow down ripening by inhibiting ethylene production in the fruit and reducing the respiration rate, can slow down the decrease in the amount of organic acid in the fruit (Patel et al., 2019). In the study, malic acid, shikimic acid, adipic acid, succinic acid, and formic acid were the main organic acids detected in pear fruit. Organic acid values except shikimic acid decreased at the end of the cold storage period. In the putrescine application, the highest organic acid content was malic acid, and the lowest was shikimic acid. Malic acid values were examined, and the fruit treated with Put + MAP had the highest content at the end of the cold storage period, followed by MAP and putrescine applications. There was no difference between the two applications, and control fruit had the lowest values. Put + MAP and putrescine applications were more effective than MAP and control applications in maintaining the amount of shikimic acid. The adipic acid values did not make a significant difference between the applications at the end of the cold storage period; putrescine applications significantly preserved the succinic acid content and there was no significant difference between the other applications. The formic acid values had significantly higher values with Put + MAP and MAP applications; there was no difference between putrescine and control applications (Table 1).

**TABLE 1:** The effect of putrescine and MAP applications on the organic acids of pear at cold storage.

Organic acid (mg 100 g^−1^)	Treatment	Storage time (day)				
		Harvest	30	60	90	120
Shikimic acid	Control	0.82	1.05 b	0.93 a	1.17 b	0.84 c
	MAP		1.57 a	0.56 a	2.01 a	0.92 c
	Putrescine		1.18 b	0.96 a	1.05 b	1.36 ab
	Put + MAP		0.70 c	0.64 a	0.93 b	1.52 a
Adipic acid	Control	23.30	28.54 b	24.46 a	16.83 b	14.24 ab
	MAP		24.09 c	21.53 a	20.45 a	14.86 ab
	Putrescine		35.60 a	21.45 a	19.32 a	18.05 a
	Put + MAP		16.08 d	14.17 b	15.30 b	14.62 ab
Malic acid	Control	522.10	836.46 a	816.14 b	472.96 ab	327.27 c
	MAP		453.00 c	937.52 a	492.27 a	431.16 b
	Putrescine		632.79 b	638.17 c	442.16 b	422.52 b
	Put + MAP		687.20 b	491.64 d	380.46 c	553.46 a
Succinic acid	Control	76.90	116.97 b	97.65 a	118.37 b	81.79 b
	MAP		168.69 a	40.21 c	211.11 a	87.02 b
	Putrescine		124.84 b	57.63 b	115.69 b	140.51 a
	Put + MAP		75.61 c	65.26 b	98.62 b	61.51 bc
Formic acid	Control	26.65	26.54 a	21.02 b	17.06 a	6.47 b
	MAP		23.97 a	23.62 a	13.69 b	9.74 a
	Putrescine		28.10 a	13.34 c	7.24 c	5.48 b
	Put + MAP		18.81 b	11.76 c	16.21 ab	10.00 a

*Note*: Means in columns with the same letter do not differ according to Tukey's test at *p* < .05.

The highest gallic acid and hydroxybenzoic acid values detected in pears were significantly maintained by Put + MAP and putrescine applications at the end of the cold storage period. Catechin values were significantly maintained with the Put + MAP application. Naringin, q‐coumaric, and quercetin values had significantly higher values with the Put + MAP application, and significant differences occurred between them and other applications. Unlike other phenolic compounds, the MAP‐applied fruit had higher values in terms of coumaric content compared to other applications (Table 2). Patel et al. (2019) reported that the decrease in acidity rate with ripening during cold storage was lower in fruit treated with spermidine, and Jongsri et al. (2017) reported that higher acidity rates were obtained during cold storage with spermidine application in mango. Kibar et al. (2021) reported that the individual phenolic contents of peaches decreased during cold storage, and the putrescine application prevented the loss of these compounds, and the effect varied depending on concentration.

**TABLE 2:** The effect of putrecine and MAP applications on specific phenolic compounds of pear at cold storage.

Phenolic compound (mg 100 g^−1^)	Treatment	Storage time(day)				
		Harvest	30	60	90	120
Gallic acid	Control	6.62	4.12 a	4.44 ab	3.90 b	4.43 b
	MAP		4.90 a	4.75 a	3.30 c	3.99 c
	Putrescine		4.45 a	4.75 a	4.55 a	5.01 a
	Put + MAP		4.60 a	3.99 b	4.14 ab	4.84 a
Catechin	Control	0.18	0.14 a	0.02 c	0.81 a	0.27 b
	MAP		0.08 b	0.02 c	0.10 c	0.24 b
	Putrescine		0.08 b	0.79 a	0.10 c	0.30 b
	Put + MAP		0.09 b	0.09 b	0.42 b	0.53 a
Hydroxybenzoic acid	Control	0.58	0.53 a	0.77 b	0.51 a	0.58 b
	MAP		0.76 a	1.42 a	0.65 a	0.58 b
	Putrescine		0.76 a	0.89 b	0.23 b	0.80 a
	Put + MAP		0.42 a	0.28 c	0.73 a	0.72 a
Ferulic acid	Control	0.33	1.35 a	0.57 a	0.03 b	0.00 a
	MAP		0.68 b	0.00 b	0.08 a	0.00 a
	Putrescine		0.36 c	0.00 b	0.00 b	0.00 a
	P + MAP		0.30 d	0.00 b	0.00 b	0.00 a
Naringin	Control	1.32	0.83 a	0.22 bc	0.52 a	0.18 c
	MAP		1.58 a	0.58 a	0.56 a	0.31 b
	Putrescine		1.20 a	0.13 c	0.11 b	0.17 c
	Put + MAP		1.85 a	0.29 b	0.24 b	0.90 a
o‐Coumaric	Control	0.40	0.23 bc	0.12 a	0.09 ab	0.00 c
	MAP		2.26 a	0.14 a	0.20 a	0.30 b
	Putrescine		0.51 b	0.09 b	0.07 b	0.20 b
	Put + MAP		0.15 c	0.13 a	0.15 a	0.50 a
Coumarin	Control	0.08	0.50 a	0.35 a	0.27 a	0.00 b
	MAP		0.26 a	0.19 ab	0.02 c	0.05 a
	Putrescine		0.20 a	0.15 b	0.17 b	0.10 b
	Put + MAP		0.25 a	0.17 b	0.04 c	0.09 b
Quercetin	Control	0.14	0.25 a	0.02 b	0.01 b	0.01 b
	MAP		0.14 b	0.05 a	0.08 a	0.02 b
	Putrescine		0.22 a	0.09 a	0.09 a	0.07 b
	Put + MAP		0.04 c	0.09 a	0.10 a	0.17 a

*Note*: Means in columns with the same letter do not differ according to Tukey's test at *p* < .05.

## 4 CONCLUSION

The weight loss, decay rate, and loss in fruit flesh firmness, which significantly affect the economic life of the fruit and consumer preference, were reduced by MAP and putrescine applications. MAP and putrescine applications delayed ripening in pear fruit, and lower SSC and higher titratable acidity values were detected in the MAP and putrescine‐applied fruit. The changes in the content of the organic acids and individual phenolics occurred during storage; they varied depending on the type of individual phenolics and organic acids. As a result, it was concluded that MAP and putrescine applications can be used effectively to delay maturity and preserve the quality of the pear fruit during cold storage.

## AUTHOR CONTRIBUTIONS

**Ferhat Ogurlu:** Conceptualization (equal); formal analysis (equal); methodology (equal); resources (equal). **Emine Kucuker:** Investigation (equal); methodology (equal); resources (equal); supervision (equal); visualization (equal). **Erdal Aglar:** Conceptualization (equal); supervision (equal); validation (equal); writing – original draft (equal); writing – review and editing (equal). **Ceyda Kizgin Ozcengiz:** Conceptualization (equal); data curation (equal); methodology (equal); resources (equal). **Cuneyt Uyak:** Data curation (equal); methodology (equal); resources (equal).

## CONFLICT OF INTEREST STATEMENT

The authors declare that there is no conflict of interests regarding the publication of this paper.

## Data Availability

All data generated or analyzed during this study are included in this published article.
